# The Evaluation of Selected Production Indicators Following the Implementation of Vaccination as Part of a BVDV Eradication Strategy in Two Endemically Infected Beef Suckler Herds

**DOI:** 10.3390/vetsci12070670

**Published:** 2025-07-16

**Authors:** Matt Yarnall, Ellen Schmitt-van de Leemput, Manuel Cerviño, Ruben Prieto, Arnaud Bolon

**Affiliations:** 1 Boehringer Ingelheim Vetmedica GmbH, Binger Strasse, 55218 Ingelheim, Germany; 2 Bovilogique, 12 Rue des Merisiers, 53700 Villaines la Juhel, France; ellen.schmitt@bovilogique.fr; 3 Boehringer Ingelheim Animal Health España, 50 Prat de la Riba, 08173 Barcelona, Spain; manuel.cervino@boehringer-ingelheim.com (M.C.); ruben.prieto@boehringer-ingelheim.com (R.P.); 4 Facultad de Veterinaria, Universidad Complutense de Madrid, 28040 Madrid, Spain; 5 Boehringer Ingelheim France, 29 avenue Tony Garnier, 69007 Lyon, France; arnaud.bolon@boehringer-ingelheim.com

**Keywords:** BVD, beef, productivity, vaccine, Bovela^®^

## Abstract

The bovine viral diarrhoea virus (BVDV) impacts the health and performance of bovine herds. In the present retrospective study, the impact of BVDV control on the production performance of suckler herds was tested by vaccinating all female cows of the herd on the same day (DV). The performance of cows in two commercial suckler herds was analysed during 12 months before DV (PREVAC, *n* = 497 cows) and 9 to 21 months after vaccination (POSTVAC, *n* = 531 cows). The proportion of calves born compared to the initial number of cows subjected to mating did not differ (PREVAC and POSTVAC, 87% and 84%, respectively). The proportion of calves weaned compared to the initial number of cows subjected to mating also did not differ between PREVAC (71%) and POSTVAC (74%). However, the proportion of calves weaned compared to the number of calves born was higher than POSTVAC (87%) when compared to PREVAC (81%). Thus, the data demonstrate that a BVDV control programme using vaccination in suckler herds can improve calf survival.

## Simple Summary

This study investigates whether vaccinating cows against a virus called bovine viral diarrhoea (BVD), which harms cattle health and productivity, could improve the performance of beef-producing herds. Researchers compared two time periods: before and after all female cows in two herds were vaccinated on the same day. They measured how many calves were born and survived to weaning. While the number of calves born and weaned stayed about the same before and after vaccination, a key improvement was seen in calf survival: more calves that were born after vaccination lived long enough to be weaned. Specifically, survival improved from 81 out of 100 calves before vaccination to 87 out of 100 calves after vaccination. This suggests that vaccinating cows against BVD can help more calves survive, which is important for farmers’ livelihoods and animal welfare.

## 1. Introduction

Bovine viral diarrhoea (BVD), caused by the BVD virus (BVDV), is endemic in many countries. The basis for seeking BVDV freedom is based on economics, welfare grounds, and proactive rather than reactive disease control with associated increased antibiotic use. Control depends on the removal of persistently infected (PI) animals and the maintenance of biosecurity to ensure that no new PIs are born. The vaccination of pregnant and future pregnant animals to prevent PI formation has been proven to be a successful biosecurity tool [1], and there are several licenced vaccines used in Europe.

BVDV control can be considered more challenging in suckler than dairy herds for several reasons. Suckler herds graze more extensively, and there is intense contact between pregnant dams and young calves, thus facilitating virus spread between PIs and pregnant cows. The motivation for farmers to eradicate BVDV is influenced by cost–benefit analyses. The literature provides multiple studies that indicate the economic benefits of BVDV eradication schemes [2,3]. However, evidence in suckler herds is more scarce than dairy herds.

Therefore, the aim of this cohort study is to add information to the existing literature on the economic benefits of BVDV control programmes in suckler herds by comparing key performance indicators (KPIs) in BVDV endemically infected beef suckler herds before and after the implementation of a BVDV eradication strategy, including vaccination with a live double-deleted BVDV vaccine (Bovela^®^, Boehringer Ingelheim, Ingelheim am Rhein, Germany). The productive performance of herds before and after the implementation of a BVDV eradication strategy, including vaccination (BESIV), will be compared using retrospective data analyses. To evaluate productive performance, several KPIs specially developed for suckler herds were used [4].

This study contributes to the understanding of the impact of endemic BVDV infections on the performance of extensive suckler herds and demonstrates the benefit of the BESIV under these conditions.

## 2. Materials and Methods

### 2.1. Ethical Approval

Ethical approval was not required as vaccination was a veterinary recommendation by the private independent veterinarians responsible for the veterinary care of the herds in question. Approval was obtained for the collection and use of data needed, as per the GDPR guidelines.

### 2.2. Study Design

**Figure 1:**
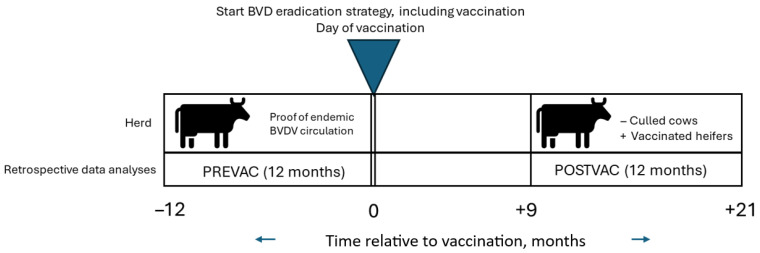
Retrospective data collection from selected herds before and after the start of the BVDV eradication strategy, including vaccination (BESIV). Herd performances were compared before and after vaccination. In herds, the initial non-vaccinated animals served as the control group for the subsequently vaccinated animals. Before vaccination (PREVAC), data were collected on the calves that were weaned during the 12 months before the day of vaccination (DV), along with data on their dams. After vaccination (POSTVAC), data were collected on the dams that calved from 9 months after the day of vaccination to 21 months after the day of vaccination. Those animals and their calves were followed up until weaning.

This is an observational, retrospective study using individual cows as the experimental unit. The performance of herds was compared before and after the BESIV. Data were collected from the periods before and after vaccination with “Bovela^®^”, as part of the BESIV (Figure 1). In herds, the initial non-vaccinated animals served as the control group for the subsequently vaccinated animals. Thus, a comparison was made between the initial herd, endemically infected with BVDV, and the same herd during the subsequent BVD-free breeding period, reduced in size by culled cows and increased in size by calving heifers. Herds were maintained under the same conditions other than the implementation of the vaccination.

Before vaccination (PREVAC), data were collected on the calves that were weaned during the 12 months before the day of vaccination (DV), along with data on their dams. After vaccination (POSTVAC), data were collected on the dams that calved from 9 to 21 months after DV. Those animals and their calves were followed until weaning. This timeframe from vaccination to protection ensured that calves were born from the protected cows.

The vaccination, a single 2 mL intramuscular injection, of all pregnant and future pregnant animals at one farm was performed on the same day, as per the recommendation of the vet according to the datasheet.

The following data were collected for the periods PREVAC and POSTVAC: the number of cows subjected to mating, the number of calves born, the date of birth of the calves, the number of calves weaned from the calvings that took place, mortality at birth, and mortality between birth and weaning. The data were summarised in frequency tables or tables with descriptive statistics as appropriate.

### 2.3. Farm Selection

Included in the study were European commercial suckler herds (from France, Spain, Ireland and the UK) endemically infected with BVDV at the start of the BESIV, recruited by independent veterinarians. For each of the herds, the proof of BVDV circulation was characterised by one of three descriptions:

- The presence of an identified PI for a minimum of six months;
- Evidence of the birth of a PI from a cow mated at the farm;
- Proof of seroconversion against BVDV during serological surveillance or other testing.

The components of the BESIV protocol, such as the diagnosis and exclusions of PIs and biosecurity measures, were the choice of the farmer in consultation with their independent veterinarian. However, for this retrospective analysis, yearly vaccination with “Bovela^®^” lyophilisate and solvent for suspension for injection for all pregnant and future pregnant animals on one certain day (DV) was a mandatory inclusion factor. Data were collected during the PREVAC and POSTVAC periods (Figure 1) from farm records and or national databases.

### 2.4. Statistical Analysis of Data

The main objective of the statistical analysis was the comparison of the performance of the herds after (POSTVAC) and before (PREVAC) the BESIV and the impact on the success or otherwise of the breeding and rearing of calves. The statistical analyses were performed using SAS software release 9.4 (SAS, 2016, Cary, NC, USA: SAS Institute Inc.).

The following variables were subjected to statistical evaluation:

The numeric production index (NPI):

$$\frac{Number\;of\;alive\;weaned\;calves}{Number\;of\;dams\;subjected\;to\;mating}\times 100\%$$

The calving rate:

$$\frac{Number\;of\;calves\;born}{Number\;of\;dams\;subjected\;to\;mating}\;\times 100\%$$

The total pre-weaning survival rate:

$$\frac{Number\;of\;alive\;weaned\;calves}{Number\;of\;calves\;born}\;\times 100\%$$

The pre-weaning survival rate of calves born alive:

$$\frac{Number\;of\;alive\;weaned\;calves}{\left(Number\;of\;calves\;born\;alive-Number\;of\;calves\;experiencing\;stillbirth\right)}\;\times 100\%$$

The following hypotheses were tested for the outcome variables:

**H0**:  *PREVAC = POSTVAC*;

**H1**:  *PREVAC* ≠ *POSTVAC*.

These tests on the differences between the study groups were designed as two-sided tests for a type I error level of α = 0.05 (5%). The data were evaluated for each country/farm separately, and for countries, pooled data were stratified by “country”. Qualitative interactions between countries were tested using the Gail–Simon test. Groups were tested on the differences for each country using Fisher’s exact test and for the pooled data using the Cochran–Mantel–Haenszel test stratified by “country”.

The target sample size was 628 animals, assuming 80% weaned control calves per mated cow, a 5% improvement following the intervention, 5% variance, 95% confidence level, and 80% power. Eight herds were sought, assuming an average herd size of 45 animals and the loss of 10% of animals’ worth of data.

## 3. Results

Initially, eight suckler herds eligible for inclusion were identified in four different European countries (France, Spain, Ireland, and the UK). The selected farms represented 928 cows in the PREVAC 12-month period (spring 2020 to 2021) and 952 cows in the POSTVAC 12-month period (winter 2021 to 2022). Unfortunately, the required complete dataset was available for only two of the farms. At six of the eight farms, the minimum data required for the females put to mating was not complete for the periods being studied, which overlapped with multiple seasonal breeding periods. However, the target sample size was exceeded due to the size of the farms recruited.

A descriptive analysis of the data is shown in Table 1. Depicted are the individual data of the two herds and then the overall data of all animals included in the study. The French herd was recruited following the detection of the birth of a PI from a dam mated at the farm, and the Spanish herd was recruited following serological surveillance detecting seroconversion (in 20 heifers); however, PIs were not actively identified. The qualitative interactions between the herds were found to be insignificant (*p* > 0.05) in all cases (Table 2). Therefore, for further analyses of the data, the overall data were used. A total number of 497 and 531 cows, in the PREVAC and POSTVAC groups, respectively, were subjected to mating and gave birth to 432 (87%, PREVAC) and 447 (84%, POSTVAC) calves: 10 (PREVAC) and 16 (POSTVAC) calves were stillborn. Of the calves that were born alive in the PREVAC period, 352 survived until weaning (83%). For the calves born alive in the POSTVAC period, this number was 391 (91%).

## 4. Discussion

In six out of the eight farms eligible for inclusion, the number of cows subjected to mating was not available; thus, reproduction parameters were unknown. Knowing that the number of cows subjected to mating is key and necessary to calculate reproduction parameters such as fertility, pregnancy, and calving rates, these parameters are important performance indicators that should guide farmers in their farm management. The limited number of herds recruited, increasing the risk of type II errors due to reduced power, should be viewed in the context of a brief report, guiding researchers to build on the outcomes presented in future studies.

In our study, there was no difference in NPI before and after the implementation of the BESIV. This KPI is constituted of two different parameters: the calving rate and the total pre-weaning survival rate. It is perhaps surprising that the calving rate did not differ. Non-successful mating can be a result of poor fertility, embryonic mortality, or abortion. Vaccination should have protected animals in the POSTVAC group from embryonic mortality or abortion due to BVDV. However, the endemic BVDV before the DV has been shown to alter the development of oocytes and impact fertility [5,6]. It is possible that the cows in the POSTVAC group still suffered from reduced ovarian function due to recent BVDV exposure. Hence, the importance of vaccination in advance of first breeding is shown.

The total pre-weaning survival rate did differ between the groups. The calf survival rate was higher in the POSTVAC group. It is possible that the differences in the number of calves weaned are due to the prevention of the birth of PIs through the use of “Bovela^®^”, but also to fewer calves dying due to secondary diseases such as scour and bovine respiratory disease (BRD) [5].

In our study, the farmers and their veterinarians chose the components of the eradication programme, which varied between herds. The removal of (4) PIs was undertaken in the French herd, along with the adoption of isolation and testing of incoming cattle. The identification and removal of PIs was not achieved in the Spanish herd, and they relied solely on vaccination with “Bovela^®^” (Boehringer Ingelheim), which was a mandatory inclusion factor for both farms. The vaccination component is important. Firstly, this is because of the study design, as the vaccination of the whole herd on DV marks a clear start to BVDV protection. However, it is also an important biosecurity measure, especially in beef farms. Suckler herds graze on widespread areas, and biosecurity boundaries are difficult to maintain. Therefore, transient or endemic infection, even after PI extinction, cannot be excluded.

While no significant changes to herd and calf management were proposed by the vet other than vaccination during this period, it is possible that seasonal variations in nutrition and weather may have confounded fertility and calf health during the study periods. This is further limited by the fact that only one calendar year was compared before and after intervention. Biosecurity practices such as the isolation and testing of incoming cattle in the French herd may have contributed to improved herd health. As a further limitation of this study, the culling of dams between the PRE and POSTVAC study groups may have removed less productive animals. Furthermore, identifying the stage of pregnancy loss through routine pregnancy diagnosis would have provided further information for evaluation.

For further research, this pilot study could be expanded to gather retrospective data from more herds or alternatively use a case–control study design to mitigate seasonal impacts. In order to predict the potential economic benefits of BVDV eradication for a suckler herd, it would be interesting to use the increased calf survival demonstrated as a parameter in an overall economic model, as proposed by numerous publications [2,7,8]. Based on this data, an increase of 6% of weaned calves at 250 kg in a 100 calved-cow herd would return EUR 6000, using local live weight prices at EUR 3.6 per kg, minus vaccination costs. To apply these models to individual farm situations, data registration should be professionalised.

To conclude, while these two herds are not representative of European suckler herds as a whole, the study suggests that the eradication of BVDV on beef suckler farms, using vaccination with a live double-deleted BVD vaccine, may increase calf survival. In order to predict the overall economic benefits of BVDV eradication, the parameter of increased calf survival could be integrated into an overall economic model.

## Figures and Tables

**Table 1:** The descriptive statistics of the productive performance data of suckler herds before and after the implementation of a BVDV eradication strategy, including vaccination. Before vaccination, (PREVAC) data were collected on the calves that were weaned during the 12 months before the day of vaccination (DV), along with the data of their dams. After vaccination (POSTVAC), data were collected on the dams that calved from nine months after the day of vaccination to 21 months after the day of vaccination. Those animals and their calves were followed up until weaning.

Country	Group	Number of Cows Subjected to Mating	Total Number of Calves Born	Number of Calves Born Alive	Number of Calves Weaned	Calving Rate % *	Total Pre-Weaning Survival Rate % **	Pre-Weaning Survival Rate of Alive-Born Calves ***	Numeric Production Index ****
France	PREVAC	161	130	126	119	81	92	94	74
	POSTVAC	192	158	142	141	82	89	99	73
Spain	PREVAC	336	302	296	233	90	77	79	69
	POSTVAC	339	289	289	250	85	87	87	74
Overall Data	PREVAC	497	432	422	352	87	81	83	71
	POSTVAC	531	447	431	391	84	87	91	74
	*p*-value for country-specific comparisons to compare heterogeneity					0.689	0.752	0.335	0.752

* Calving rate: (the number of calves born/number of cows subjected to mating) × 100. ** Total pre-weaning survival rate: (the number of calves weaned/number of calves born) × 100. *** Pre-weaning survival rate of alive-born calves: (the number of calves weaned/(number of calves born − number of calves experiencing stillbirth)) × 100. **** Numeric production index: (the number of calves weaned/number of cows subjected to mating) × 100.

**Table 2:** The frequency tables and results of tests of the productive performance data of suckler herds before and after the implementation of the BVDV eradication strategy, including vaccination.

Numeric Production Index *		***N* Weaned**	**% Weaned**	**95% CI**		***N* Matings**	***p***
	PREVAC	352	71	66.6	74.8	497	0.328 (0.460)
	POSTVAC	391	74	69.7	77.3	531	
Calving Index **		*N* born	% born	95% CI		*N* matings	
	PREVAC	432	87	83.6	89.8	497	0.250
	POSTVAC	447	84	80.8	87.2	531	(0.355)
**Total Pre-Weaning** Survival Rate ***		*N* weaned	% weaned	95% CI		*N* born	
	PREVAC	352	81	77.5	85.0	432	0.0223 * (0.254)
	POSTVAC	391	87	84.0	90.4	447	
**Pre-Weaning Survival Rate** of Alive-Born Calves ****		*N* weaned	% weaned	95% CI		*N* born alive	
	PREVAC	352	83	79.5	86.8	422	0.00230 ** (0.500)
	POSTVAC	391	91	87.6	93.3	431	

N: number of animals; CI: confidence interval. *p*: *p*-value of Cochran–Mantel–Haenszel test (‘pooled”) of the comparison between treatment groups, *p*-value of the Gail–Simon test on qualitative interactions in brackets. ns: not significant, *p* > 0.05; *: significant, *p* < 0.05; **: significant, *p* < 0.01. * Numeric production index: (the number of calves weaned/number of cows subjected to mating) × 100. ** Calving rate: (the number of calves born/number of cows subjected to mating) × 100. *** Total pre-weaning survival rate: (the number of calves weaned/number of calves born) × 100. **** Pre-weaning survival rate of alive-born calves: The number of calves weaned alive/(number of calves born − number of calves experiencing stillbirth) × 100.

## Data Availability

The data are available to reviewers upon request.

## Abbreviations

The following abbreviations are used in this manuscript:

BVDV	Bovine viral diarrhoea virus
BESIV	BVDV eradication strategy including vaccination
DV	Day of vaccination
KPI	Key performance indicator
NPI	Numeric production index
PREVAC	Data collection period (12 months before DV) of the animals before vaccination
POSTVAC	Data collection period (9 to 21 months after DV) of the animals after vaccination
